# Comprehensive analysis of risk factors associating with Hepatitis B virus (HBV) reactivation in cancer patients undergoing cytotoxic chemotherapy

**DOI:** 10.1038/sj.bjc.6601699

**Published:** 2004-03-09

**Authors:** W Yeo, B Zee, S Zhong, P K S Chan, W-L Wong, W M Ho, K C Lam, P J Johnson

**Affiliations:** 1 Department of Clinical Oncology, Sir Y.K. Pao Centre for Cancer, Chinese University of Hong Kong, Prince of Wales Hospital, Shatin, NT, Hong Kong; 2 Department of Microbiology, Sir Y.K. Pao Centre for Cancer, Chinese University of Hong Kong, Prince of Wales Hospital, Shatin, NT, Hong Kong

**Keywords:** hepatitis B virus reactivation, chemotherapy, risk factors

## Abstract

For cancer patients with chronic hepatitis B virus (HBV) infection, who receive cytotoxic chemotherapy, HBV reactivation is a well-described complication, which may result in varying degrees of liver damage. Several clinical features and the pre-chemotherapy HBV viral load have been suggested to be associated with an increased risk of developing the condition: (1) to assess the clinical and virological factors in a comprehensive manner and thereby identify those that are associated with the development of HBV reactivation; (2) to develop a predictive model to quantify the risk of HBV reactivation. In all, 138 consecutive cancer patients who were HBV carriers and undergoing chemotherapy were studied, of which 128 patients had sera available for real-time PCR HBV DNA measurement. They were followed up throughout their course of chemotherapy and the HBV reactivation rate was determined. The clinical and virological features between those who did and did not develop viral reactivation were compared. These included age, sex, baseline liver function tests, HBeAg status and viral load (HBV DNA) prior to the chemotherapy, and the use of specific cytotoxic agents. In all, 36 (26%) developed HBV reactivation. Multivariate analysis revealed pre-chemotherapy HBV DNA level, the use of steroids and a diagnosis of lymphoma or breast cancer to be significant factors. Based on real-time HBV DNA PCR assay, detectable baseline HBV DNA prior to the administration of cytotoxic chemotherapy, the use of steroids and a diagnosis of lymphoma or breast cancer are predictive factors for the development of HBV reactivation. A predictive model was developed from the current data, based on a logistic regression method.

For cancer patients who have chronic hepatitis B virus (HBV) infection, there is a high rate of hepatic complications during cytotoxic chemotherapy, and this has mainly been attributable to HBV reactivation. The condition is manifested with abnormal liver function tests that show a hepatitic picture, and it is confirmed by raised levels of serum HBV DNA. The clinical spectrum ranges from asymptomatic hepatitis to fatal hepatic failure (Galbraith *et al*, 1975; Hoofnagle *et al*, 1982; Lok *et al*, 1991; Nokamura *et al*, 1996; Kumagai *et al*, 1997; Markovic *et al*, 1999; Yeo *et al*, 2000a). However, even in its mildest form with spontaneous recovery, a patient's prognosis from cancer may still be impaired from the disruption in chemotherapeutic administration with treatment delay, or premature termination of the anticancer therapy. The incidence of HBV reactivation in hepatitis B surface antigen (HBsAg) seropositive cancer patients undergoing cytotoxic chemotherapy has been reported to be 20% or higher (Lok *et al*, 1991; Nokamura *et al*, 1996; Kumagai *et al*, 1997; Markovic *et al*, 1999; Yeo *et al*, 2000a). No preventive measures have been proven to prevent or reduce the incidence of HBV reactivation, although more recent reports have suggested that the prophylactic use of the antiviral agent lamivudine, prior to the start of chemotherapy, may reduce the occurrence of the condition (Rossi *et al*., 2001; Liao *et al*, 2002; Lim *et al*, 2002; Persico *et al*, 2002; Shibolet *et al*, 2002; Yeo *et al*, 2002). There has also been concern about the emergence of viral mutant as a result of lamivudine therapy, and, to date, limited data are available on the clinical impact of these mutants in immunosuppressed subjects. Limiting the use of the prophylactic antiviral to patients who are at the highest risk of developing viral reactivation may reduce the potential complication of mutant emergence, and at the same time be more cost-effective.

Despite the wide recognition of HBV reactivation, there is still no consensus as to the associated risk factors. Clinical features such as young age, male sex, the diagnosis of lymphoma and the use of anthracyclines and/or steroids as part of the anticancer therapy have been suggested (Lok *et al*, 1991; Yeo *et al*, 2000a), while virological factors such as HBeAg positivity (Yeo *et al*, 2000a, 2000b) and, more recently, the pre-chemotherapy HBV viral load (Lau *et al*, 2002) have also been associated.

The objectives of this study were: (1) to assess the clinical and virological factors in a comprehensive manner in order to identify those that are associated with the development of HBV reactivation; and (2) to develop a predictive model to identify patients more likely to develop HBV reactivation.

## PATIENTS AND METHODS

In all, 138 consecutive cancer patients who were chronic HBV carriers and planned for cytotoxic chemotherapy were consented and followed up throughout their course of treatment. The study was approved by the Clinical Research Ethics Committee of the Chinese University of Hong Kong.

### Investigations

Prior to study entry, the following investigations were undertaken in all patients: hepatitis B s-antigen (HBsAg), hepatitis B e-antigen/antibody (HBeAg/anti-HBe) HBV DNA level (measured by commercially available Quantiplex HBV DNA assay (bDNA), Chiron, USA), complete blood picture (CBP, which included haemoglobin, red cell count, mean cell volume, white cell count and differential and platelet count), renal function tests (RFT, which included sodium, potassium, urea, creatinine and creatinine clearance), liver function test (LFT, which included total protein, albumin, total bilirubin, alanine transaminase, alkaline phosphatase) and clotting profile. In addition, one 3-ml serum sample was collected for later retrospective analysis of HBV DNA by a real-time PCR assay (Zhong *et al*, in press).

During the course of chemotherapy, on days 1 and 10 of each cycle, CBP, clotting profile, RFT and LFT were monitored together with clinical signs and symptoms. Monitoring of these parameters was continued for 8 weeks after completion of chemotherapy.

When a patient was found to have developed hepatitis (as defined below) during the course of chemotherapy, HBV DNA was performed (measured by Quantiplex bDNA assay), with immunoglobulin M (IgM) antibody to hepatitis A virus (IgM anti-HAV), HCV RNA, anti-HDV, ANA and other investigations as clinically indicated.

### Hepatitis serology

Hepatitis A, B and D markers were detected by commercial enzyme immunoassays (Cobas Core Anti-HAV IgM EIA, Roche Diagnostics GmbH, Deutschland; Auszyme MC Dynamic (HBsAg), Abbott Laboratories, USA; Cobas Core Anti-HBc IgM EIA, Roche Diagnostics GmbH, Deutschland; MONOLISA HBe, Sanofi Diagnostics Pasteur, USA; Abbott Anti-Delta EIA, Abbott Laboratories, USA). HCV RNA was detected by reverse transcription polymerase chain reaction using primers 209, 211, 939 and 940 as previously described (Chan *et al*, 1992).

For routine use including establishment of the diagnosis of HBV reactivation, HBV DNA level was measured by using the branched DNA hybridisation assay (Quantiplex HBV DNA assay (bDNA), Chiron, USA), which has a lower detection limit of 0.7 × 10^6^ genome equivalent ml^−1^.

### Real-time HBV DNA PCR assay

For the purpose of assessing the impact of viral load on HBV reactivation, we used the real-time PCR to quantify serum HBV DNA, which has a lower detection limit for HBV DNA of 2.9 × 10^3^ g.e. ml^−1^ (Zhong *et al*, in press). In total, 128 patients had sera available for real-time PCR analysis and these were tested in a single batch. Their serum samples were separated as soon as the blood was coagulated and stored at −80°C in RNase-free tubes. Isolation of nucleic acids was performed using the High Pure Viral Nucleic Acid Kit (Roche, Mannheim, Germany). For each isolation, 200 *μ*l of serum was used resulting in 50 *μ*l of nucleic acid extract. For HBV DNA quantification by real-time PCR, PCR amplification was performed with PCR primers located at regions that are universally conserved among the six HBV genotypes (A–F) corresponding to the HBV X gene. The oligonucleotide sequences of primers were: txs3 (1434–154), TCT CAT CTG CCG GAC CGT GT; xas1 (1668–148), AAT TTA TGC CTA CAG CCT CC. A volume of 5 *μ*l equivalent of serum was used for real-time amplification in a final 10 *μ*l reaction volume, using 1 × Fast Start SYBR Green I Master Mix (Roche), MgCl_2_ (4 mM) and 0.3 *μ*M concentration of each primer. The amplification procedure, utilising the LightCycler instrument (Roche), was as follows: 95°C for 10 min, followed by 45 cycles of 95°C for 15 s, 57°C for 10 s and 72°C for 12 s, followed by denaturation of amplification samples by slow increase of temperature (0.1°C s^−1^) up to 95°C.

### Definition of HBV reactivation

The following definitions, based on a definition of Lok *et al* (1991), and subsequently modified by us (Yeo *et al*, 2000a), were applied. ‘Hepatitis’ was defined as a threefold or greater increase in serum ALT level that exceeded the reference range (>58 iu l^−1^) or an absolute increase of ALT to over 100 iu l^−1^. ‘HBV reactivation’ was defined as either one of the following: using the Quantiplex b DNA assay, Chiron assay, an increase in HBV DNA levels of 10-fold or greater when compared with the baseline level, or an absolute increase of HBV DNA level that exceeded 1000 × 10^6^ g.e. ml^−1^ during chemotherapy, in the absence of clinical or laboratory features of acute infection with hepatitis A, C and delta virus or other systemic infections.

### Comprehensive assessment of the clinical and virological factors in association with the development of HBV reactivation

Among the 128 patients who had sera available for the real-time PCR analysis for HBV DNA, the following factors were compared between those who did and those who did not develop viral reactivation: age, sex, tumour type, use of steroids, anthracyclines and 5-fluorouracil, pre-chemotherapy liver functions (albumin, total bilirubin and alanine transaminase), HBeAg status, HBV DNA using real-time PCR measurement.

### Statistical methods

Univariate analysis for detecting significant prognostic factors for HBV reactivation was done using *χ*^2^ test or Fisher's exact test. Prognostic factors for HBV reactivation were then determined by multivariate analysis using a stepwise logistic regression model based on a backward elimination procedure for model selection, with a significance level of <0.1 for a factor to enter into the logistic model; the same significance level was used to retain variables in the final model. A predictive model was obtained using the parameters of the final logistic model. The classification to the HBV reactivation group was based on the probability estimate from the logistic model, where an optimal cutoff point was determined using the ROC curve technique; the ROC curve refers to the receiver operating characteristic curve, which is a plot of the true-positive rate *vs* false-positive rate associated with the classification rules for all possible choices of critical values (Hanley, 1989).

## RESULTS

In all, 36 patients were determined to have developed HBV reactivation (the ‘reactivation’ group) and 92 patients were determined not to have HBV reactivation (the ‘non-reactivation’ group) (Table 1

**Table 1:** Characteristics of the 128 HBsAg seropositive cancer patients undergoing cytotoxic chemotherapy

	**Patients who had hepatitis B viral reactivation during chemotherapy**	**Patients who did not develop hepatitis B viral reactivation during chemotherapy**	***P*-value**
Total no. of patients	36	92	
			
Sex*			
Male	16 (28.6%)	40 (71.4%)	1.0000
Female	20 (27.8%)	52 (72.2%)	
			
Median age (years)	46.5	49	0.2654
Range	30–71	20–78	
			
Tumour type*			
Breast cancers	16 (41.0%)	23 (59.0%)	
Lymphomas	7 (58.3%)	5 (41.7%)	
Gastrointestinal cancers	2 (6.9%)	27 (93.1%)	0.0041
Head and neck cancers	5 (29.4%)	12 (70.6%)	
Lung cancers	3 (23.1%)	10 (76.9%)	
Other cancers	3 (16.6%)	15 (83.4%)	
			
*Pretreatment (baseline) biochemistry*			
Median ALT levels (normal <58 iu l^−1^)	35 (range: 12–77)	27 (range: 10–96)	0.1025
Median total bilirubin levels (normal <15 *μ*mol l^−1^)	7 (range: 2–26)	7 (range: 1–108)	0.9639
Median albumin levels (normal >40 g l^−1^)	35 (range: 22–43)	36 (range: 20–47)	0.8421
			
*Use of anthracyclines*			
Yes	19 (46.3%)	22 (53.7%)	0.0029
No	17 (19.5%)	70 (80.5%)	
			
*Use of steroids*			
Yes	22 (39.3%)	34 (61.2%)	0.0174
No	14 (19.4%)	58 (80.6%)	
			
*Use of fluorouracil*			
Yes	14 (23.0%)	47 (77.0%)	0.2419
No	22 (32.8%)	45 (67.2%)	
			
HBeAg status*			
Positive	4 (26.7%)	11 (73.3%)	1.0000
Negative	32 (28.3%)	81 (71.7%)	
			
HBV DNA-real-time PCR assay*			
Detectable	31 (37.8%)	51 (62.2%)	0.0010
Undetectable	5 (10.9%)	41 (89.1%)	

* Fisher's exact two-sided test.

).

There were 56 males and 72 females; 16 (28.6%) males and 20 (27.8%) females developed HBV reactivation (*P*=1.000). Between the ‘reactivation’ and the ‘non-reactivation’ groups, the median ages were 46.5 and 49.0 years, respectively (*P*=0.265). There were 39 patients with breast cancer, 29 with gastrointestinal malignancies, 17 head and neck cancers, 13 lung cancers, 12 non-Hodgkin's lymphoma and 18 other malignancies. Among these patients, 16 who had breast cancers (41.0% of the patients with this tumour type, *P*=0.053), seven non-Hodgkin's lymphoma (58.3%, *P*=0.037), two gastrointestinal malignancies (6.9%, *P*=0.0041), five head and neck (29.4%, *P*=1.000), three lung cancers (23.1%, *P*=1.000) and three (16.6%, *P*=0.396) with other malignancies developed viral reactivation.

Of the 128 patients, 15 were HBeAg positive, of which four (26.7%) developed reactivation, while 32 of the 113 (28.3%) patients who were HBeAg negative/anti-HBe positive developed the condition (*P*=1.000). Using the real-time PCR assay, 82 patients were detected to have HBV DNA, 31 (37.8%) of whom developed reactivation; in contrast, only five of the 41 (10.9%) patients who had undetectable HBV DNA developed the condition (*P*=0.001).

There was no statistically significant difference in baseline liver function in terms of ALT, bilirubin and albumin between the two groups of patients. Out of 56 patients, 22 (39%) received steroids and developed reactivation; while 14 of 72 patients who did not receive the agent developed the condition (*P*=0.017). Out of 41 patients, 19 (46.34%) received anthracyclines and developed reactivation, while 17 of the 87 (19.54%) patients who had no anthracyclines developed the condition (*P*=0.0029). With respect to 5-fluorouracil, 61 received the drug, among whom 14 (23%) developed reactivation, while 22 of 67 patients (33%) who did not receive the agent developed the condition (*P*=0.2419).

On multivariate analysis, the factors that were significantly associated with a higher risk of developing HBV reactivation were detectable HBV DNA levels based on real-time PCR measurement (*P*=0.0003, Odds ratio of 8.4 with 95% CI from 2.6 to 27.2), the use of steroid (*P*=0.0465, OR of 2.7 with 95% CI from 1.0 to 7.2), a diagnosis of lymphoma (*P*=0.0419, OR of 5.0 with 95% CI from 1.1 to 23.5) and breast cancer (*P*=0.0041, OR of 4.2 with 95% CI from 1.6 to 11.0).

### Predictive model development

A predictive model was determined using the final logistic regression model, as shown in Table 2

**Table 2:** Logistic regression model

				**95% confidence interval**	
	**Parameter**	***P*-value**	**Odds ratio**	**Lower**	**Upper**
Intercept	−3.6589 (*β*_0_)				
Lymphoma (x1)	1.6086 (*β*_1_)	0.0419	5.0	1.06	23.53
Breast (x2)	1.4264 (*β*_2_)	0.0041	4.2	1.57	11.02
Steroid (x3)	0.9939 (*β*_3_)	0.0465	2.7	1.02	7.19
HBV PCR (x4)	2.1339 (*β*_4_)	0.0003	8.4	2.63	27.15

**Figure 1:**
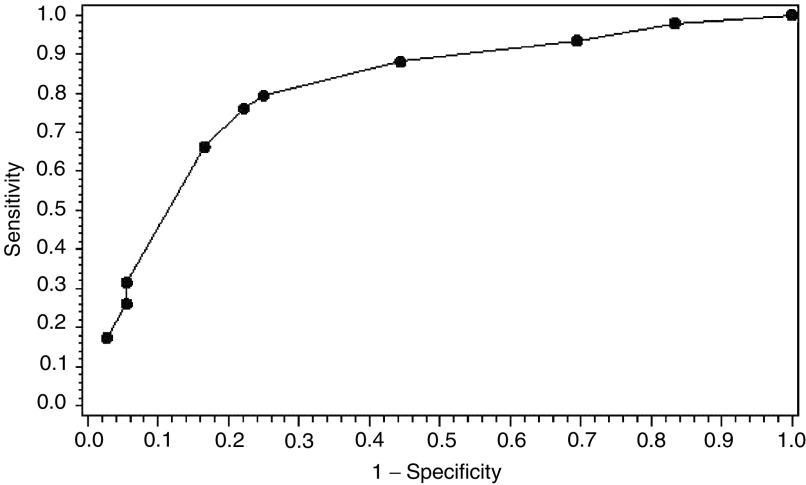
Logistic model with PCR_HBV, steroid, lymphoma and breast sites.

. The following is the mathematical formula that can be programmed into a personal computer or programmable calculator:


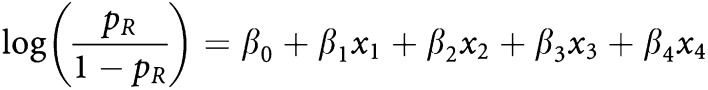


where *p*_R_ is the probability of HBV reactivation, *x*_1_, *x*_2_, *x*_3_ and *x*_4_ are indicator variables for the presence of lymphoma (presence=1, absence=0), breast cancer (presence=1, absence=0), the use of steroids (presence=1, absence=0) and HBV virology load determined by PCR (detectable=1; not detectable=0). The parameters *β*_0_, *β*_1_, *β*_2_, *β*_3_, *β*_4_ are parameters whose values are given in Table 2. We would classify a patient to the group with a higher likelihood of developing HBV reactivation if the estimated *p*_R_ from the above logistic model is higher than 0.3. A sensitivity of 75% and a specificity of 79.3% were obtained based on a cutoff point of 0.3 for the predictive probability of the logistic model. This optimal predictive probability cutoff point was determined using the ROC curve method (Figure 1

).

## DISCUSSION

Hepatitis B virus reactivation during cytotoxic chemotherapy is a particularly important clinical issue in areas of the world such as China, where chronic HBV infection is endemic. With the increasing incidence of neoplastic diseases (Lok *et al*, 1991), and the more widespread use of cytotoxic chemotherapy, the occurrence of HBV reactivation is likely to increase further.

In this study, the identified risk factors included detectable pre-chemotherapy HBV DNA load (using real-time PCR measurement), the use of steroids, a diagnosis of lymphoma or breast cancer, another suggested factor being the use of anthracyclines. Previous studies have reported factors including male sex (Liaw *et al*, 1987; Liang *et al*, 1990; Lok *et al*, 1991), younger age (Yeo *et al*, 2000a), HBeAg positivity (Lok *et al*, 1991; Yeo *et al*, 2000a, 2000b) and the presence of lymphoma (Yeo *et al*, 2000a). Although the reason remains unknown, male sex has consistently been reported to be a risk factor for the exacerbation of chronic HBV infection in cancer patients undergoing chemotherapy, as well as in non-cancer subjects (Liaw *et al*, 1987; Liang *et al*, 1990; Lok *et al*, 1991). Although HBeAg positivity in immunocompromised cancer patients appears to be a risk factor for HBV reactivation (Liang *et al*, 1990; Yeo *et al*, 2000b), this has not been found to be universally the case (Lok *et al*, 1991; Nokamura *et al*, 1996), and an increased risk may be partly attributed to the presence of the pre-core/core promoter HBV mutant (i.e. HBeAg negative/anti-HBe positive), which had been associated with severe fulminant hepatitis (Omata *et al*, 1991; Ehata *et al*, 1993; Liang *et al*, 1994; Nokamura *et al*, 1996; Steinberg *et al*, 2000; Yeo *et al*, 2000a, 2000b). In addition, consistent with other reports, the baseline (pretreatment) liver function including ALT, total bilirubin and albumin levels did not appear to be associated with the development of HBV reactivation.

Certain limitations are noted in this study. Of the 82 patients found to have detectable HBV DNA on real-time PCR assay, 67 were HBeAg seronegative, and the existence of precore mutants was not analysed. In addition, the diagnosis of HBV reactivation based on HBV DNA measurement upon the development of hepatitis could be suboptimal in two aspects. First, the commercially available assay (Quantiplex bDNA assay) used was relatively less sensitive, with a lower detection limit of 0.7 × 10^6^ g.e. ml^−1^). Secondly, in the absence of serial monitoring HBV DNA, the true incidence of viral reactivation might have been underestimated, as the rise in HBV DNA might have preceded overt hepatitis in some cases (Yeo *et al*, 2001).

Several chemotherapeutic agents have been reported to be associated with the development of HBV reactivation in cancer patients. Apart from steroids and anthracyclines, other drugs that have been reported included vincristine, bleomycin, etoposide, methotrexate, actinomycin D, mercaptopurine, azauridine, chlorambucil, cytosine arabinoside, leucovorin, cisplatin and gemcitabine (Galbraith *et al*, 1975; Hoofnagle *et al*, 1982; Thung *et al*, 1985; Bird *et al*, 1989; Lau *et al*, 1989; Liang *et al*, 1990; Pinto *et al*, 1990; Lok *et al*, 1991; Soh *et al*, 1992; Nokamura *et al*, 1996; Wong *et al*, 1996; Yeo *et al*, 2000a). In the present study, it is interesting to find that the use of steroids was an associated risk factor on multivariate analysis. Patients with lymphoma have more frequently been reported to develop HBV reactivation (Lok *et al*, 1991; Omata *et al*, 1991; Nokamura *et al*, 1996; Yeo *et al*, 2000a), and a recent report on breast cancer patients receiving chemotherapy has shown the viral reactivation rate to be as high as 41% (Yeo *et al*, 2003). The type of treatment and the type of tumour may be inter-related factors. Since disease site confounded with the use of certain cytotoxic agents, a separate stepwise logistic regression model without disease site was used for further analysis. The logistic model without considering the disease site revealed that anthracyclines (*P*=0.0662, OR 2.44, 95% CI from 0.94 to 6.32), as well as steroids (*P*=0.0557, OR 2.56, 95% CI from 0.98 to 6.69) and detectable HBV DNA (*P*=0.0011, OR of 6.25, 95% CI from 2.08 to 18.8) were significant factors. Steroids and anthracyclines are commonly used as part of the cyclophosphamide/adriamycin/vincristine/prednisolone (CHOP) regimen for patients with non-Hodgkin's lymphoma; they also constitute adriamycin/cyclophosphamide (AC) combination chemotherapy commonly used for breast cancer patients (where steroid is generally administered in the anti-emetic pre-medication). Indeed, anthracyclines have mainly been administered to patients with non-Hodgkin's lymphoma and breast cancer (Table 3

**Table 3:** Tumour type treated with anthracyclines

	**No. of patients with the tumour type**	**No. of patients treated with anthracyclines**
Breast cancers	39	22 (56%)
Lymphomas	12	10 (83%)
Gastrointestinal cancers	29	0
Head and neck cancers	17	0
Lung cancers	13	0
Other cancers	18	9 (50%)

). Although it is generally accepted that CHOP is an immunosuppressive regimen, only 4% of Western breast cancer patients receiving AC developed severe immunosuppression (Fisher *et al*, 1990). However, recent data from Chinese breast cancer patient population revealed the corresponding figure to be as high as 77% (Ma *et al*, 2002). This implies that the degree of immunosuppression as a result of the treatment could have been a more significant factor than the type of malignancy *per se* in the development of HBV reactivation. This is further supported by one of the findings from the present study that revealed that patients with gastrointestinal malignancies, who undergo cytotoxic chemotherapy mainly consisting of less immunosuppressive agents (fluorouracil and folinic acid), have a lower risk of developing viral reactivation. Previous study has suggested that the type of chemotherapeutic regimens, rather than the individual cytotoxic agent, may be a more important factor (Yeo *et al*, 2000a). Thus, when the use of 5-fluorouracil was analysed in the present study, no association was found with HBV reactivation; while the agent was commonly coupled with folinic acid in gastrointestinal malignancies, it was also often used in combination with other cytotoxics that resulted in a variable degree of immunosuppression for non-gastrointestinal malignancies.

Using the real-time PCR assay, which has a lower cutoff value of 2.9 × 10^3^ g.e. ml^−1^, the present study has revealed that detectable HBV DNA load prior to chemotherapy was a significant predictive factor for viral reactivation. Although a recent study has demonstrated that a detectable HBV DNA may predict HBV reactivation in HBsAg-positive individuals (Lau *et al*, 2002), the finding, which was based on a small heterogeneous group of patients who underwent autologous hematopoietic cell transplantation, could not be generalised and applied to the majority of cancer patients who require conventional cytotoxic chemotherapy without transplantation.

Prophylactic treatment with antiviral nucleotide analogue lamivudine prior to the start of chemotherapy has been suggested to be effective in reducing the incidence of HBV reactivation (Rossi *et al*, 2001; Liao *et al*, 2002; Lim *et al*, 2002; Persico *et al*, 2002; Shibolet *et al*, 2002; Yeo *et al*, 2002). The use of the proposed predictive model may aid the identification of high-risk patients who stand to benefit most from the antiviral, which could in turn be administered in a most cost-effective manner.

We conclude that, in cancer patients who are positive for HBsAg and who undergo cytotoxic chemotherapy, high HBV viral load (>2.9 × 10^3^ g.e. ml^−1^) prior to the administration of cytotoxic chemotherapy is a significant predictive factor for the development of HBV reactivation. Other factors that have been identified include the use of steroids and a diagnosis of lymphoma and breast cancer. The combination of baseline HBV viral load (using real-time PCR) and clinical features provide a more sensitive and specific means of identifying patients who are at risk of developing HBV reactivation, and who would benefit most from lamivudine prophylaxis. Although a predictive model has been formed to identify patients who are at risk of developing HBV reactivation in this study, there is a need to validate it in a separate cohort of patients before it can be safely applied in the clinical practice.
